# Glycosylated Lipopeptides—Synthesis and Evaluation of Antimicrobial Activity and Cytotoxicity

**DOI:** 10.3390/biom13010172

**Published:** 2023-01-13

**Authors:** Karol Sikora, Marta Bauer, Sylwia Bartoszewska, Damian Neubauer, Wojciech Kamysz

**Affiliations:** Department of Inorganic Chemistry, Faculty of Pharmacy, Medical University of Gdańsk, 80-416 Gdansk, Poland

**Keywords:** ultrashort cationic lipopeptides, glycosylation, *N*-acetyl-d-glucosamine, synthesis, ESKAPE, antibacterial, antifungal, antibiofilm, hemolysis, cytotoxicity

## Abstract

Ultrashort cationic lipopeptides (USCLs) are promising antimicrobial agents that may be used to combat pathogens such as bacteria and fungi. USCLs consist of a few basic amino acid residues and at least one lipid moiety, usually a fatty acid chain. Generally, USCLs are potent antimicrobials but their major shortcoming is a relatively high cytotoxicity and hemolytic activity. Glycopeptide antibiotics (e.g. vancomycin) are essential in combating bacterial infections and are popular in medicinal practice. However, literature concerning the effect of glycosylation of peptides on their antimicrobial activity is rather scarce. For the first time, this study highlights the effect of USCLs glycosylation on in vitro biological activity. The aim of this study was to evaluate the impact of glycosylation of a series of USCLs on antimicrobial activity, cytotoxicity and hemolytic activity. Straight-chain fatty acids (C14, C16, C18) were attached to the *N*-terminal amino group of tripeptides—SRR-NH_2_, RSR-NH_2_ and RRS-NH_2_. Two groups of the lipopeptides were synthetized, the first with unmodified L-serine (USCLs) and the other with L-serine *O*-glycosylated by *N*-acetyl-β-d-glucosamine to produce new class of glycosylated ultrashort cationic lipopeptide (gUSCLs). Both USCLs and gUSCLs were tested against planktonic and biofilm cultures of ESKAPE strains (*Enterococcus faecium*, *Staphylococcus aureus*, *Klebsiella pneumoniae*, *Acinetobacter baumannii*, *Pseudomonas aeruginosa*, *Enterobacter* spp.) and *Candida glabrata*, and hemolytic activity on human erythrocytes and cytotoxicity against the HaCaT cell line was examined. Generally, USCLs and gUSCLs proved to be active against all the tested strains. The highest activity displayed was by lipopeptides containing the C18 fatty acid. Antimicrobial, hemolytic and cytotoxic activities were mainly correlated with amino acid sequence (position of serine/glycosylated serine) and hydrophobicity of molecule and were found to be highly strain-dependent. In general, glycosylation did not guarantee an increased antimicrobial activity or a decreased hemolytic and cytotoxic activities. However, in some cases, gUSCLs proved to be superior to their USCLs analogs. The most pronounced differences were found for peptides with C18 fatty acid and serine at the first and second position against both planktonic cells and biofilm of *C. glabrata*, as well as the second and third position against *S. aureus*. It is noteworthy that gUSCLs were also more active against biofilm than were USCLs.

## 1. Introduction

In the light of an increasing microbial resistance and the insufficient development of new drugs, there is an urgent need for novel antimicrobials. The WHO and EMA have unanimously supported activities for the research and development of antibiotics in order to combat multidrug-resistant pathogens [1,2]. Ultrashort cationic lipopeptides (USCLs) are promising candidates for new antimicrobial drugs owing to their extraordinary antibacterial and antifungal activities. USCLs are peptides that contain only a few amino acid residues, mainly basic ones, and a lipid chain that is usually a straight-chain fatty acid conjugated to *N*_α_-amino group of the *N*-terminal amino acid residue. Unfortunately, USCLs have also been known as substantially cytotoxic and hemolytic compounds [3,4,5]. They interact with the cell membrane, permeabilize it and induce uncontrollable leakage of ions and intramolecular substances, leading to the cell death [6]. Therefore, further studies on USLCs have been focused on modifications that can increase cell specificity. High antimicrobial activity and selectivity of some peptides can be attributed to their glycosylation. For instance, formaecin I (isolated from red bulldog ant, *Myrmecia gulosa*) and M-drosocin (isolated form fruit fly, *Drosophila melanogatser*) are naturally occurring glycosylated antimicrobial peptides (gAMPs). Studies confirmed that *O*-glycosylation plays a pivotal role in their antimicrobial activity [7,8]. Usually, serine, threonine, asparagine or cysteine bear a sugar in peptides which can be *N*-, *O*-, *S*-, or even *C*- glycosylated [9]. There is a variety of sugar moieties that were found in glycosylated AMPs, e.g. glucose, mannose, glucosamine, *N*-acetyl-glucosamine, galactosamine and *N*-acetyl-galactosamine [10,11,12,13]. It was reported that glycosylation could improve antimicrobial activity, stability, target specificity, and contribute to affect host immune system [14]. Hypothetically, conjugation of peptides with sugars can bring new molecules to effectively combat microbial infections. Sugar residues are rich in hydroxyl groups that can possibly interact with pathogen membrane phospholipids and/or membrane proteins facilitating USCLs–cell surface interactions and therefore elevate membrane perturbations. Talat et al. [8] have modified formaecin I and M-drosocin with different sugar moieties. It is worthwhile to note that peptides with β-d-GlcNAc exhibited an advantage over non-glycosylated ones in terms of their antibacterial activity. Furthermore, *N*-acetyl-d-glucosamine was recognized as being able to bind specifically to some bacterial lectins, inhibiting pathogen cell adhesion to human tissues and therefore preventing further colonization [15]. The aim of this study was to evaluate the effect of USCLs glycosylation with the *N*-acetyl-β-d-glucosamine (β-d-GlcNAc) residue on antimicrobial and hemolytic activities and cytotoxicity to human keratinocytes. According to our knowledge, such studies have never been conducted on USCLs to date.

## 2. Materials and Methods

### 2.1. Synthesis

#### 2.1.1. Synthesis of Fmoc-Ser(ß-d-GlcNAc(Ac)_3_)-OH

*N*-(9-Fluorenylmethoxycarbonyl)-(2-acetamido-2-deoxy-3,4,6-tri-*O*-acetyl-β-d-glucopyranosyl)-L-serine was synthesized according to the method reported by Carvalho et al. [16] with modifications. 2-Acetamido-2-deoxy-3,4,6-tri-*O*-acetyl-α-d-glucopyranosyl chloride [17] (365 mg, 1 mmol) dissolved in 5 mL of 1,2-dichloroethane, was placed in the round-bottom flask dropwise, over 15 min, a mixture of *N*-(9-fluorenylmethoxycarbonyl)-l-serine (164 mg, 0.5 mmol) and mercuric bromide (296 mg, 1.1 mmol) in 1,2-dichloroethane (5 mL), was added. The mixture was heated for 1 h at 80 °C, followed by the addition of 2-acetamido-2-deoxy-3,4,6-tri-*O*-acetyl-α-d-glucopyranosyl chloride (365 mg, 1 mmol) in 5 mL of 1,2-dichloroethane, and the mixture was heated for an additional 1 h. It was then diluted in 20 mL DCM and washed three times with 10 mL of 3% EDTA. The organic layers were combined, dried over MgSO_4_ and concentrated after purification with RP-HPLC on a Phenomenex Gemini-NX C18 column (21.20 × 100 mm, 5.0 µm particle size, 110 Å pore size). UV detection at 280 nm and gradient elution with a linear 10–70% acetonitrile gradient in deionized water over 60 min at room temperature were used. The mobile phase flow rate was 20.0 mL/min. Acetonitrile and water, both containing 0.1% of TFA, were used as a mobile phase. The purity and identity of the product were confirmed with LC-MS analysis using Waters Alliance e2695 system with Waters 2998 PDA and Acquity QDA detectors (software—Empower®3 Waters, Milford, MA, USA) on a Waters XBridge™ Shield RP-18 column (4.6 × 150 mm, 3.5 µm particle size, 130 Å pore size. ESI-MS: *m*/*z* found: 657.47 [M + H]^+^ theoretical *m*/*z* 657.22 calculated for C_32_H_37_N_2_O_13_)). The structure of the product was confirmed with ^1^H, NMR analysis, on Bruker AVANCE III (Bruker, Billerica, MA, USA), at 500 MHz in CDCl_3_: CD_3_OD (1:1) mixture as solvent. ^1^H NMR: 7.77 (2 H, d, *J* 7.5, Fmoc Ph), 7.65 (2 H, dd, *J* 3.4 Hz, *J* 7.3 Hz Fmoc Ph), 7.43 (2 H, t, *J* 7.2 Hz Fmoc Ph), 7.31 (2 H, t, *J* 7.5 Hz Fmoc Ph), 5.22 (1 H, t, *J_2_*_,*3*_ 10.3, H-3), 5.00 (1 H, t, *J_3_*_,*4*_ 9.8, H-4), 4.69 (1 H, d, *J_1_*_,*2*_ 8.5, H-1), 4.44 (1 H, dd, *J* 10.6, *J* 6.5, CH_2a_ Fmoc), 4.40 (1 H, t, *J* 4.2 CH Ser), 4.35 (1 H, dd, *J* 10.4, *J* 7.0, CH_2b_ Fmoc), 4.28–4.21 (2 H, m, H-6, CH Fmoc), 4.17 (1 H, dd, *J* 10.8, *J* 4.6, CH_2a_ Ser), 4.11 (1 H, dd, *J_5_*_,*6′*_ 2.0, *J_6_*_,*6′*_ 12.2, H-6′), 3.91 (1 H, dd, *J* 10.7, *J* 3.8, CH_2b_ Ser), 3.83 (1 H, dd, *J_1_*_,*2*_, *J_2_*_,*3*_, H-2), 3.73 (1 H, ddd, *J_4_*_,*5*_ 10.8, *J_5_*_,*6*_ 4.4, H-5), 2.06, 2.02, 2.00, 1.87 (12 H, 4 × s, COCH_3_).

#### 2.1.2. Synthesis of Lipopeptides

All lipopeptides were synthesized manually using the solid-phase peptide synthesis (SPPS) method and Fmoc chemistry on polystyrene resin modified by a Rink amide linker. Deprotection of the Fmoc groups was carried out in a 20% (*v*/*v*) piperidine (Merck, Darmstadt, Germany) solution in DMF (Honeywell, Seelze, Germany) for 15 min at room temperature. Attachment of the protected amino acids was conducted in a DMF/DCM solution (Chempur, Piekary Slaskie, Poland) with coupling agents using a threefold molar excess of DIC (Peptideweb, Zblewo, Poland) and OxymaPure (Iris Biotech GmbH, Marktredwitz, Germany) for 1.5 h at room temperature. All reactions were performed using a Kamush peptide shaker (Kamush, Poland). Every step was preceded by rinsing the resin with DMF and DCM. Chloranil test was used for control of acylation and deprotection processes. The peptides were cleaved from the resin using one of the mixture of TFA, TIS, and water (95:2.5:2.5 *v*/*v*). Crude products were precipitated with cold diethyl ether (Chempur, Piekary Slaskie, Poland) and lyophilized.

#### 2.1.3. Removal of Acetyl-protecting Groups of Glucosamine Residues in Lipopeptides

Removal of acetyl-protecting groups from the sugar residue in lipopeptides (10–18) was carried out according to Jensesn et al. [18] The peptide was dissolved in dry MeOH (2 mL) and 0.1 M NaOMe in MeOH was added dropwise until a pH of 10 was reached. After 15 min of stirring under nitrogen, the reaction was quenched through the addition of solid CO_2_. Then, the solvent was evaporated and the product was purified by means of RP-HPLC.

#### 2.1.4. Purification of Lipopeptides and Glycolipopeptides

Purifications were carried out on a Phenomenex Gemini-NX C18 column (21.20 × 100 mm, 5.0 µm particle size, 110 Å pore size). UV detection at 214 nm was used, and the crude peptides were eluted with a linear 10–70% acetonitrile gradient in deionized water over 90 min at room temperature. The mobile phase flow rate was 10.0 mL/min. Acetonitrile and water, both containing 0.1% of TFA, were used as a mobile phase. The purity and identity of the peptide was confirmed with LC-MS analysis. The RP-HPLC system was used—Waters Alliance e2695 system with Waters 2998 PDA and Acquity QDA detectors (software—Empower®3). All analyses were carried out on a Waters XBridge™ Shield RP-18 column (4.6 × 150 mm, 3.5 µm particle size, 130 Å pore size). Samples (10 µL) were analyzed with a linear 10–90% acetonitrile gradient in deionized water over 15 min at 25.0 ± 0.1 °C. The mobile phase flow rate was 0.5 mL/min. Both eluents contained 0.1% (*v*/*v*) of formic acid. Mass analysis and UV detection at 214 nm were used. Pure fractions (>95%, by HPLC analysis) were collected and lyophilized.

#### 2.1.5. Determination of Peptide Hydrophobicity with RP-HPLC

To determine peptide hydrophobicity, a Waters Alliance e2695 system with a Waters 2998 PDA Detector was used. All analyses were carried out on a Waters X-Bridge Shield RP-18 column (3.0 × 100 mm, 3.5 μm particle size, 130 Å pore size). The peptides were dissolved in water (0.1% TFA, *v*/*v*) up to a concentration of 1 g/L. UV detection at 214 nm was used, and samples (10 μL) were eluted with a linear 20–80% acetonitrile gradient in deionized water over 30 min at 25.0 ± 0.1 °C. The mobile phase flow rate was 0.5 mL/min. Both eluents contained 0.1% (*v*/*v*) of TFA. Each peptide sample was analyzed in triplicate. Maximum standard deviation and coefficient of variation were 0.046 and 0.23%, respectively.

### 2.2. Antimicrobial Activity

#### 2.2.1. Cultivation of Microorganisms

The *Acinetobacter baumannii* ATCC BAA-1605, *Enterococcus faecium* ATCC 700221, *Klebsiella aerogenes* ATCC 13048, *Klebsiella pneumoniae* ATCC 700603, *Pseudomonas aeruginosa* ATCC 9027, *Staphylococcus aureus* ATCC 33591, *Candida glabrata* ATCC 15126, strains were acquired from the American Type Culture Collection (ATCC). All the strains were stored at −80 °C in Roti-Store cryo vials and were transferred into fresh Mueller–Hinton broth (MHB, Becton Dickinson, Warsaw, Poland) for bacteria or RPMI-1640 (Sigma-Aldrich, Steinheim, Germany) for fungi before the tests and incubated for 24 h at 37 °C. Then, the cultures were seeded on the Mueller–Hinton agar (BioMaxima, Lublin, Poland) or Sabouraud dextrose agar (SDA, BioMaxima, Lublin, Poland) plates, respectively, and incubated as just mentioned. These agar cultures were used for further microbiological assays. Cell densities for all assays were adjusted spectrophotometrically (Multiskan GO Microplate Spectrophotometer, Thermo Fisher Scientific, Vantaa, Finland) at 600 and 530 nm for bacteria and fungi, respectively.

#### 2.2.2. Activity against Planktonic Cultures

The MICs were determined using a broth microdilution method according to the Clinical and Laboratory Standard Institute guidelines [19,20]. For this purpose, initial inoculums of bacteria (5 × 10^5^ CFU/mL) in MHB and yeasts (2 × 10^3^ CFU/mL) in RPMI-1640 with 2% d-glucose were exposed to the ranging concentration of lipopeptides (1–512 μg/mL) and incubated at 37 °C for 18 h and 24 h, respectively. The experiments were conducted on 96-well microtiter polystyrene plates (Kartell, Italy). The growth was assessed visually after incubation and the MIC was assumed as the lowest peptide concentration at which a noticeable growth of microorganisms was inhibited. All experiments were conducted in triplicate.

#### 2.2.3. Activity against Biofilm

The MBECs were determined as previously described, using 96-well polystyrene flat-bottom plates [21,22]. For this purpose, 24 h cultures of microorganisms were diluted to obtain a final density of 5.0 × 10^5^ CFU/mL and 2.0 × 10^5^ CFU/mL of bacteria and fungi, respectively. Microorganisms were diluted in MHB or RPMI-1640 with 2% d-glucose (bacteria and fungi, respectively). Briefly, 100 µL of cell suspension was added into the test plates. After 24 h of incubation at 37 °C, the wells were rinsed three times with a phosphate buffer saline (PBS, pH 7.4) to remove non-adherent cells. Subsequently, 100 μL of the test compounds over the concentration range (1–512 μg/mL) were added to each well. After 24 h of incubation at 37 °C, 20 μL of a cell viability reagent was added (resazurin, 4 g/L; Sigma Aldrich, St. Louis, MO, USA). The MBEC was read after 1 h. The determined values were recorded as the lowest concentration at which the reduction of resazurin (from blue to pink) was visible. All experiments were conducted in triplicate.

### 2.3. Hemolytic Activity

The hemolysis assay was conducted using a procedure described previously [23]. For this purpose, fresh human red blood cells (RBCs) with EDTA anticoagulant were washed three times with phosphate-buffer saline (PBS) using centrifugation at 800× *g* for 10 min and resuspended in PBS. Then, the stock solution of RBCs was added to serial dilution of peptides on 96-well polystyrene plates to reach a final volume of 100 µL with 4% concentration of erythrocytes (*v*/*v*) over the concentration range of 1–512 µg/mL of the tested compounds. The control wells for the 0% and 100% hemolysis consisted of RBCs suspended in PBS and 1% of Triton X–100, respectively. Subsequently, the plates were incubated for 60 min at 37 °C and then centrifuged at 800× *g* for 10 min at 4 °C (Sorvall ST 16R Centrifuge, Thermo Scientific, Osterode am Harz, Germany). After centrifugation, the supernatant was carefully resuspended to new microtiter plates and the release of hemoglobin was monitored through absorbance measurements at 540 nm (Multiskan™ GO Microplate Spectrophotometer, Thermo Scientific). All experiments were conducted in triplicate.

### 2.4. MTT Assay

To evaluate the cytotoxicity of the test peptides (IC_50_), the classic MTT assay on 96-well plates was performed for human keratinocytes (HaCaT) which were acquired from the ATCC. The assay was based on colorimetric determination of the cell metabolic activity where the color intensity reflected the number of live cells that could be measured spectrophotometrically. The cell line was cultured in a Dulbecco’s modified Eagle Medium (Invitrogen) supplemented with 10% fetal bovine serum (*v*/*v*), 100 units/mL of penicillin, 100 μg/mL of streptomycin, and 2 mM L-glutamine and was kept at 37 °C in a humidified 5% CO_2_ incubator. Briefly, a day after plating of 500 cells per well, a series of concentrations (0.5–500 μg/mL) of the test compounds were applied. DMSO was added to the control cells at a final concentration of 1.0% (*v*/*v*), which was related to the maximum concentration of the solvent compounds used in the experiment. After 24 h of incubation at 37 °C (humidified 5% CO_2_ incubator) with the peptides, a medium containing 1 mg/mL of MTT was added to the wells up to a final concentration of 0.5 mg/mL. Subsequently, the plates were incubated at 37 °C for 4 h. Then, the medium was aspirated, and the formazan product was solubilized with DMSO. The background absorbance at 630 nm was subtracted from that at 570 nm for each well (Epoch, BioTek Instruments, Winooski, VT, USA). Six replicates were conducted for each concentration. All experiments were repeated at least twice and the resulting IC50 values were calculated with a GraFit 7 software (v. 7.0, Erithacus, Berkley, CA, USA).

## 3. Results and Discussion

### 3.1. Synthesis

**Scheme 1:**
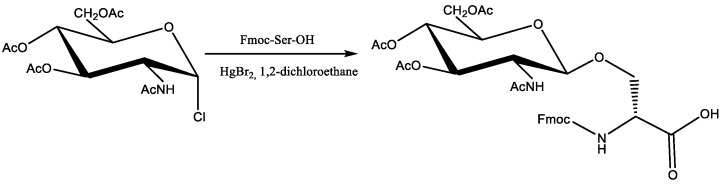
Synthesis of *N*-(9-fluorenylmethoxycarbonyl)-(2-acetamido-2-deoxy-3,4,6-tri-O-acetyl-β-d-glucopyranosyl)-l-serine.

The C16-RR-NH_2_ an USCL with a well-documented antimicrobial activity was used as a parent molecule for designing a new class of glycosylated ultrashort cationic lipopeptides (gUSCL). Our previous studies indicate that amino acid position and fatty acid chain length are crucial to keep the balance between hydrophobicity and hydrophilicity of the molecule. These are significant features that can affect biological activity [22]. In general, two main groups of USCLs were studied—basic lipopeptides and their analogs with an *O*-glycosylated serine residue. The straight-chain fatty acids (C14, C16, C18) were conjugated to *N^α^*-amino group of *N*-terminal amino acid residue. Serine/glycosylated serine residue were placed in three different positions—“SRR”, ”RSR”, and ”RRS”. In effect, each group contained 9 compounds (18 in total). USCLs were synthesized using the solid phase and Fmoc/tBu chemistry. For the synthesis of gUSCLs, *N*-(9-fluorenylmethoxycarbonyl)-(2-acetamido-2-deoxy-3,4,6-tri-*O*-acetyl-β-d-glucopyranosyl)-l-serine (Fmoc-Ser(ß-d-GlcNAc(Ac)_3_)-OH) was synthetized using the method reported by Carvalho et al. [16] with slight modifications. To a solution of 2-acetamido-2-deoxy-3,4,6-tri-*O*-acetyl-α-d-glucopyranosyl chloride [17] in 1,2-dichloroethane, Fmoc-Ser-OH and HgBr_2_ in 1,2-dichloroethane was added dropwise. Then, the mixture was heated at 80 °C for 2 h. Additional portion of 2-deoxy-3,4,6-tri-*O*-acetyl-α-d-glucopyranosyl chloride was added after 1 h. After purification through extraction and reverse phase high-performance liquid chromatography (RP-HPLC), the product was obtained in satisfactory yield (ca. 50%). The reaction was repeated several times to provide a sufficient amount of the product (Scheme 1). Its structure was confirmed with MS and ^1^HNMR analyses. The results were consistent with those reported by Carvalho et al. [16], including β configuration of anomeric carbon atom with a coupling constant *J_1–2_* of 8.5 Hz.

**Scheme 2:**
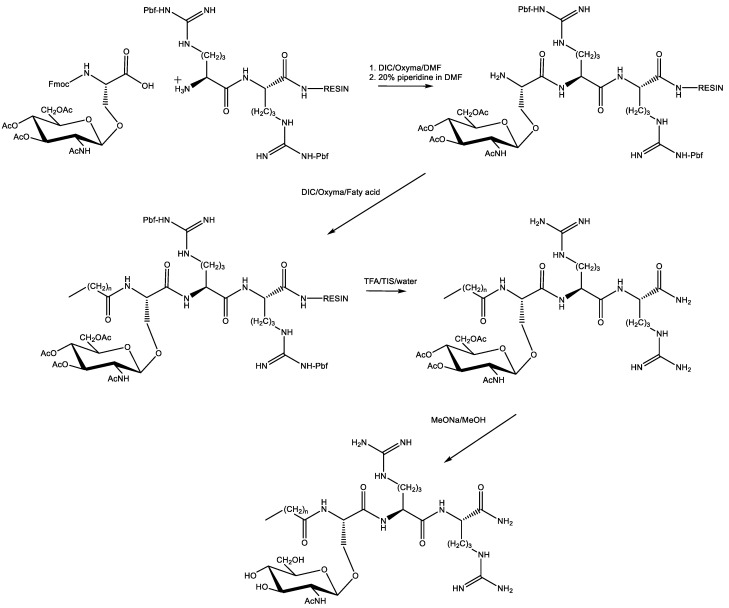
General procedure for the synthesis of glycolipopeptides **10**–**18**; {*n* = 12, 14, 16}.

Fmoc-Ser(ß-d-GlcNAc(Ac)_3_)-OH was used to synthesize gUSCLs by means of peptide synthesis on the solid support. After cleavage of gUSCLs, an additional synthesis step was performed to remove *O*-acetyl protecting groups from the *N*-acetyl-glucosamine residue. To do this, the crude peptide was dissolved in MeOH and sodium methanolate was added dropwise. After 15 min, dry ice was added to quench the reaction. Finally, after evaporation of the solvent, all gUSCLs were purified by RP-HPLC. A general procedure for the synthesis of glycolipopeptides is presented in Scheme 2.

After synthesis and purification, all the products were analyzed with MS and HPLC to confirm purity and identity of the compounds. Amino acid sequences, results of MS analyses and adjusted retention time of the compounds are displayed in Table 1. The chromatographic analyses were performed with RP-HPLC; retention time refers to hydrophobicity of the lipopeptide.

**Figure 1:**
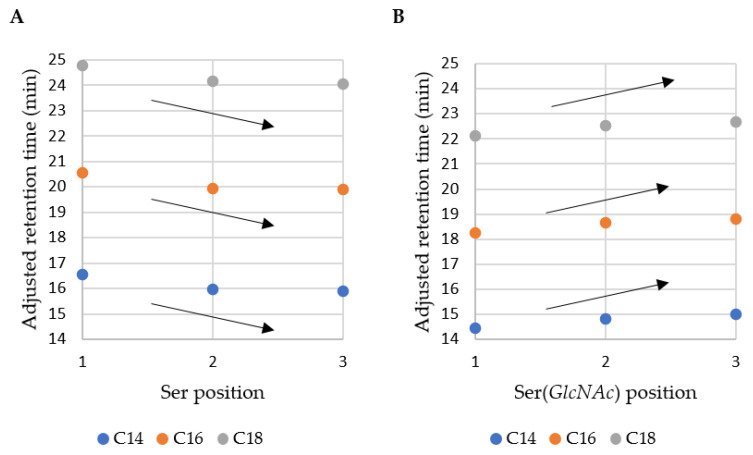
Adjusted retention time of lipopeptides vs. position of (**A**) Ser and (**B**) Ser(*GlcNAc*).

The adjusted retention time was analyzed and the results of the correlation between position of serine/glycosylated serine, fatty acid chain length and retention time are shown in Figure 1. It can be deduced that non-glycosylated lipopeptides are more hydrophobic than their glycosylated analogs (gUSCLs; higher retention times). This can be explained by the presence of three hydroxyl groups and the amide bond that can be regarded as hydrophilic components due to their tendency to form hydrogen bonds. In the case of all fatty acids (C14, C16, C18), the hydrophobicity of USCLs decreases with an increasing distance between the fatty acid residue and the sugar moiety. This finding is consistent with our previous results, where USCL with *N*-terminal phenylalanine (C16-FRR-NH_2_) was the most hydrophobic and the *C*-terminal one was the least hydrophobic (C16-RRF-NH_2_) [22]. During RP-HPLC analysis, the molecules interact with alkyl chains (C18) of stationary phase. Hypothetically, interactions between the hydrophobic region of USCL and the stationary phase should not be perturbed by hydrophilic moieties in order to be effective. The serine residue contains a hydroxyl group but also the methylene group and a C_α_ atom that can interact with alkyl chains. On the other hand, gUSCLs displayed an opposite pattern—compounds with “S(*GlcNAc*)RR-NH_2_” are the most hydrophilic and those with “RRS(*GlcNAc*)-NH_2_” are the most hydrophobic. This can explained by the hydrophilic character of the sugar entity that can be repulsed from C18 stationary phase. Moreover, the sugar moiety adjacent to fatty acid residue can hypothetically disrupt effective interactions between the alkyl chains.

### 3.2. Antimicrobial Activity against Planktonic Cultures

Antibacterial and antifungal activity of compounds were determined using serial microdilution method against reference strains of ESKAPE group: *Acinetobacter baumannii*, *Enterococcus faecium*, *Klebsiella aerogenes*, *Klebsiella pneumoniae*, *Pseudomonas aeruginosa*, *Staphylococcus aureus*, and one fungus: *Candida glabrata*. Minimum inhibitory concentration of the lipopeptides are shown in Table 2.

A high antimicrobial activity of USCLs against *E. faecium* (MIC of 8 µg/mL) with one exception (compound **1**, 16 µg/mL) was noticed. The results for their glycosylated analogs showed a slightly lower activity, with MIC ranging from 8 to 32 µg/mL. However, enhanced activity with elongation of the hydrocarbon chain length in fatty acid residue and the change of serine position to 2 or 3 could be seen. Furthermore, USCLs were very active against *S. aureus* with MIC 8–16 µg/mL. Their glycosylated counterparts were less active, with MIC of 16 µg/mL for **13**–**18** and 32–64 µg/mL for **10**–**12**. Generally, the low antimicrobial activity of the lipopeptides was noticed against *K. pneumoniae* and *K. aerogenes*, while only compounds with C18 fatty acid residues (**7**–**9**, **16**–**18**) displayed a moderate activity with MIC of 16–64 µg/mL. It is noteworthy that gUSCLs **16**–**18** with C18 fatty acid showed a higher activity against *A. baumannii* and *P. aeruginosa* compared with that of their non-glycosylated analogs (**7**–**9**). In addition, peptides **7**–**9** and **16**–**18** were most active against *C. glabrata* with MIC of 4 µg/mL.

Analyses of retention time and MIC (log_2_MIC) revealed that in some cases, there was a linear correlation between them (Figures S1–S7). The only exceptions were USCLs in combination with *S. aureus*, *E. faecium*, *A. baumannii*, and *P. aeruginosa*. This can be explained in terms of comparable antimicrobial activity of the compounds. A moderate linear correlation was found with USLCs and *K. pneumoniae*, as well as between gUSCLs and *E. faecium* and *S. aureus* (R^2^ between 0.5982 and 0.6452). Again, a strong correlation was found with USCLs and *K. aerogenes* (R^2^ = 0.7155) and *C. glabrata* (R^2^ = 0.8722) as well as between gUSCLs and *K. pneumoniae* (R^2^ = 0.7055), *A. baumannii* (R^2^ = 0.7923), *P. aeruginosa* (R^2^ = 0.7454), *K. aerogenes* (R^2^ = 0.7254), and *C. glabrata* (R^2^ = 0.9779). A correlation between antimicrobial activity and lipophilicity (adjusted retention time) of organic compounds including USCLs has been reported [22,24]. In general, the most hydrophobic lipopeptides have the lowest MIC values. Usually, glycosylation leads to compounds with a decreased antimicrobial activity with some exceptions (bolded values in Table 2). This was found in Gram-negative strains—*K. pneumoniae*, *A. baumannii*, and *P. aeruginosa*. Six out of the seven cases include compounds with the most hydrophobic C18 alkyl chain. No pattern was observed; however, second position is the only one that glycosylated gave compounds with enhanced antimicrobial activity to against all three of the strains just mentioned. Importantly, C18-S(*GlcNAc*)RR-NH_2_ and C18-RS(*GlcNAc*)R-NH_2_ have the lowest MIC against *A. baumannii* (16 µg/mL) among all of the tested compounds.

### 3.3. Antimicrobial Activity against Biofilm

Lipopeptides with octadecanoic acid resides (C18; USCLs **7**–**9** and gUSCLs **16**–**18**) were selected for studies on biofilm owing to their highest activity against planktonic cultures. Minimum biofilm eradication concentrations of the compounds are presented in Table 3.

In general, gUSCLs displayed an enhanced antibiofilm activity compared to that of USCLs parent molecules. Interestingly, this finding can be attributed to lipopeptides with *C*-terminal serine (**9** vs. **18**) residue in the case of all the tested bacterial strains. The MBEC values are significantly higher than those of MIC due to a specific biofilm structure and its natural resistance to antibiotics. This result is compatible with that of our previous studies on USCLs and biofilm. Usually, MBEC of USCLs are a few times higher than their MIC [21,25,26]. gUSCLs (**16**–**18**) were slightly more active to *S. aureus* and *K. pneumoniae* biofilms (128–256 µg/mL) than those of *A. baumannii* and *P. aeruginosa* (≥256 µg/mL). Our previous studies on USCLs indicated that *S. aureus* ATCC 33591 biofilm could be eradicated at substantially lower concentrations than biofilm of Gram-negative strains (*K. pneumoniae*, *A. baumannii*, *P. aeruginosa*), e.g., MBECs of C12-RR-NH_2_ were 64 and 512 µg/mL, respectively [25]. Interestingly, biofilms of *S. aureus* and *K. pneumoniae* showed a similar susceptibility to gUSCLs. The lowest MBEC (64 µg/mL) values were obtained against *C. glabrata* for compounds **16** and **17** (64 µg/mL). Moreover, they were more effective against biofilm than their non-glycosylated analogs.

### 3.4. Hemolysis of Human Erythrocytes

The lipopeptides were tested against human erythrocytes in order to evaluate their hemolytic activity. The results are presented in Table 4. Moreover, selectivity indexes (SI) were calculated by dividing the HC_50_ by MIC. SI higher than 1 indicates that a compound can be selective, while values lower than 1 indicate nonselective compounds. In general, with an increase in alkyl chain length, HC_50_ decreases, indicating that those compounds are more hemolytic. Comparisons of HC_50_ for USCLs and gUSCLs failed to provide a clear picture of glycosylation effect on hemolytic activity. In some cases, glycosylation led to suppressed toxicity, e.g., a comparison of C14 peptides analogs: **1**–**3** and **10**–**12**. On the other hand, an increase in toxicity of C18 analogs (**7**–**9** vs. **16**–**18**) was found for glycosylated USCLs. For this reason, SI was calculated to provide a correlation between antimicrobial activity and toxicity. The most selective lipopetides were USCLs **7**–**9** with high SI values (>10) against *E. fecium*, *S. auresus* and *C. glabrata*. Among gUSCLs: **10** and **11** with C14 fatty acid and **16**–**18** should be highlighted owing to their high selectivity against *E. fecium* and *C. glabrata.* The values of SI above 10 were bolded in Table 4.

**Figure 2:**
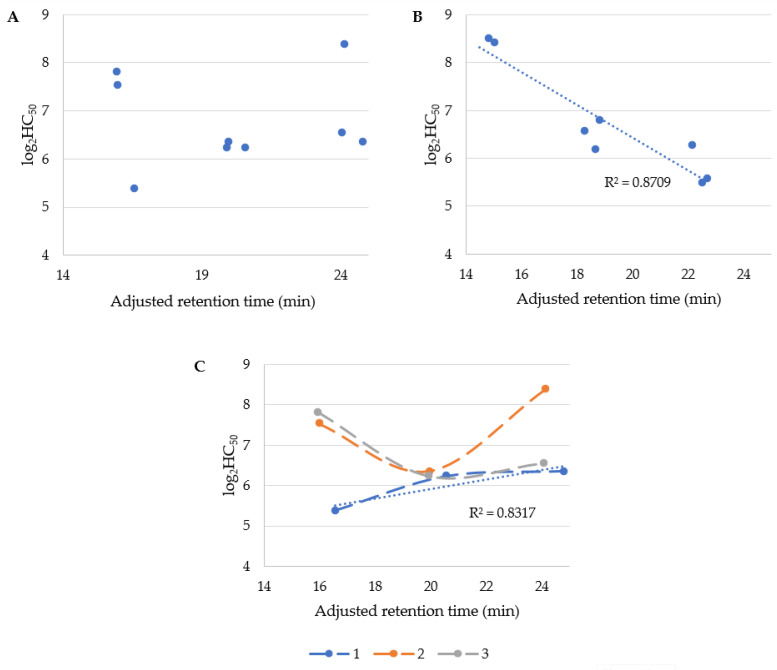
Adjusted retention time vs. log_2_HC_50_ of (**A**) USCLs and (**B**) gUSLCs, and (**C**) USCLs differentiated into three series depend on serine position.

Furthermore, hemolytic activity (HC_50_) was correlated with USCLs hydrophobicity (retention time). The results are presented in Figure 2. It can be concluded that HC_50_ of USCLs is not correlated with their hydrophobicity (Figure 2A). This could be quite surprising since biological activity of membrane-active compounds depends, to some extent, on their hydrophobicity [27,28,29]. It seems likely that the position of serine residue in the case of this series of USCLs is crucial to hemolytic activity. When the serine is located at first position in USCLs, their hemolytic activity (log_2_HC_50_) is linearly correlated with the adjusted retention time (R^2^ of 0.8317). It can be deduced that hemolysis caused by compounds of this particular series is decreasing with the elongation of the lipid tail. USCLs with the serine residue at the second and third position are similarly hemolytic and in both series, lipopeptide with hexadecenoic acid (C16) has the highest hemolytic potential. On the other hand, hemolytic activity of gUSCLs is linearly correlated with their hydrophobicity—R^2^ = 0.8709 (Figure 2B). Moreover, selectivity indexes were also correlated with the adjusted retention time (Figures S8–S14). With USCLs, *K. pneumoniae* and *C. glabrata*, there was a moderate linear correlation (R^2^ of 0.6446 and 0.6539, respectively) and a substantially linear one for *K. aerogenes* (R^2^ of 0.8235). Selectivity indexes of HC_50_ of USCLs and their MIC against *S. aureus*, *E. faecium* and *K. aerogenes* exhibited a noticeably linear correlation (R^2^ between 0.5843 and 0.6433) but significantly linear in terms of *K. pneumoniae* and *C. glabrata* (R^2^ of 0.7622 and 0.8693, respectively). No such a pattern was found for the remaining strains and HC_50_-related SIs vs. the adjusted retention time.

### 3.5. Cytotoxicity against HaCaT Cell Line

Cytotoxicity against immortalized human keratinocytes (HaCaT cell lime) was evaluated and the results are presented in Table 5 (IC_50_). The selectivity indexes were calculated for all the strains as IC_50_/MIC. According to IC_50_, in most cases, glycosylation caused only a slight decrease in toxicity. However, in contrast with SI calculated for HC_50_, the differences between USCLs and gUSCLs are not so distinct. Generally, SI did not exceed the value of 1; only in the case of the few ones could a value above 1 be noticed (bolded values in Table 5). Moreover, toxicity of USCLs and gUSCLs is high and exceeds their antimicrobial activity. The lipopetides were not selective against Gram-negative strains (*K. pneumoniae*, *A. baumannii*, *P. aeruginosa*, *K. aerogenes*) over human cells. A few USCLs were selective (SI > 1) against *E. faecium* and *S. aureus* with SI up to 1.87; and some gUSCLs were selective against *E. faecium* but not against *S. aureus*, with SI between 0.63 and 1.73. Those with octadecanoic acid (C18) used in this study exhibited a distinctive selectivity against *C. glabrata*. In this case, SI of gUSCLs was noticeably higher than that of corresponding parent USCLs (2.07, 1.99, 2.67 vs. 2.53, 2.36, 3.46, respectively). Selectivity indexes were plotted against adjusted retention time (Figures S15–S21). A moderate linear correlation was found in USCLs and *K. pneumoniae*, *C. glabrata*, and gUSCLs and *K. pneumoniae*, *K. aerogenes* (R^2^ between 0.6064 and 0.6481). A relatively high linear correlation was obtained for USCLs and K. aerogenes (R^2^ of 0.8214) and also for gUSCLs and *C. glabrata* (R^2^ of 0.9223).

**Figure 3:**
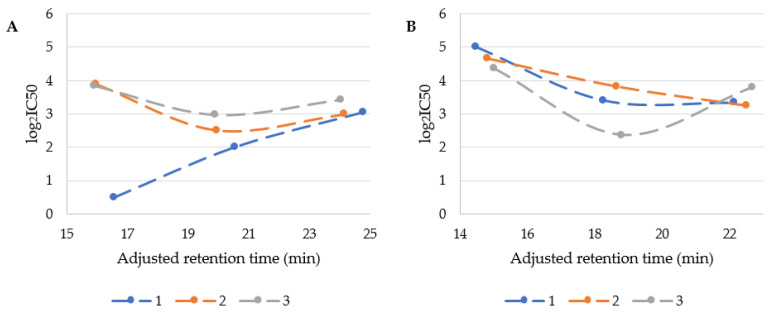
Cytotoxicity (log_2_IC_50_) of USCLs (**A**) and gUSCLs (**B**) vs. the adjusted retention time.

In Figure 3, the IC50 vales were plotted against adjusted retention time.

It is probable that cytotoxicity depends not only on the lipopeptides’ hydrophobicity but also on serine residue position. USCLs with serine at first position were usually the most cytotoxic and their log_2_IC_50_ were linearly correlated with retention time (R^2^ of 0.9852). Interestingly, an increasing lipophilicity of this group of USCLs resulted in reduced cytotoxicity (Figure 3A, blue series—1). The remaining two series of USCLs (serine at the second or third position) displayed tendencies similar to each other, while compounds with hexadecanoic acid residues (C16) were the most cytotoxic. These results are compatible with those obtained for hemolytic activity (Figure 2C). Generally it can be deduced that the cytotoxicity of USCLs decreases with changing serine position (1 < 2 < 3). The citotoxicity of gUSCLs was generally lower than that of the parent molecules (Figure 3A vs. Figure 3B). The log_2_IC_50_ of gUSCLs with serine at the first and second position are similar and both are linearly correlated with retention time (R^2^ of 0.7763 and 0.9877, respectively)—increased hydrophobicity resulted in elevated cytotoxicity. The remaining series with serine at third position displayed a different pattern than that of the first two, but is rather similar to the series of parent USCLs with serine at second and third position, where lipopeptide with hexadecanoic acid is the most cytotoxic. A linear correlation for this series was missing. The decreasing cytotoxicity with an increasing lipophilicity found in some series can be explained with aggregation. It is known that USLCs are prone to form micelles and aggregates. It was demonstrated that an increasing aggregate size could reduce interactions with biological membranes and, consequently, their cytotoxicity [27,30].

## 4. Conclusions

In this study, two series of ultrashort cationic lipopeptides containing fatty acids (C14, C16, C18) attached to the *N*-terminal amino group of tripeptides—SRR-NH_2_, RSR-NH_2_ and RRS-NH_2_—were synthesized. In the first group, unmodified serine was applied, while in the other, a side chain of serine was *O*-glycosylated by attachment of *N*-acetyl-β-d-glucosamine residue was used. Both USCLs (9 lipopeptides) and gUSCLs (9 lipopeptides) were tested for their antimicrobial activity against ESKAPE strains (*Enterococcus faecium*, *Staphylococcus aureus*, *Klebsiella pneumoniae*, *Acinetobacter baumannii*, *Pseudomonas aeruginosa*, and *Enterobacter* spp.) and *Candida glabrata* using determination MIC and MBEC (activity against planktonic and biofilm forms, respectively). Moreover, the toxicity of the compounds was assessed against erythrocytes and HaCaT cell line. The highest antimicrobial activity was found for USCLs and gUSCLs containing stearic acid (C18) residue. Interestingly, *E. fecium* was the most susceptible to lipopeptides and the second in line was *S. aureus* with MIC of 8–32 and 8–64 µg/mL, respectively. Usually, glycosylation led to compounds with a decreased antimicrobial activity, with a few exceptions of Gram-negative strains—*K. pneumoniae*, *A. baumannii*, and *P. aeruginosa*.

In general, gUSCLs were slightly more active against biofilm than their non glycosylated counterparts. Lipopeptides containing C18 fatty acid proved to be most effective against *C. glabrata* planktonic and biofilm cultures with a slightly higher activity of glycosylated analogs with serine at the first and second position in the sequence. The lipopeptides turned out to be toxic to HaCaT cell lines with slightly lowered cytotoxicity of gUSCLs. In some cases, correlation between selectivity indexes and retention time was noticed. Moreover, antimicrobial, cytotoxic and hemolytic activities seem to be correlated with hydrophobicity and position of serine/glycosylated serine in the amino acid sequence. Generally, glycosylation of peptides had an effect on enhanced antimicrobial activity and decreased toxicity in some cases. The results clearly indicate that this type of peptide modification should be considered individually and there is no straightforward correlation between glycosylation and antimicrobial, cytotoxic and hemolytic activities. Our observations are consistent with those reported in [31]. Presumably, carbohydrates other than *N*-acetyl-β-d-glucosamine are likely to trigger a more significant change in activity.

## Figures and Tables

**Table 1:** Sequences, MS analysis and retention times of lipopeptides **1**–**18**.

Peptide	Sequence	MS Analysis			Adjusted Retention Time (min)
		*z* ^a^	*m/z* ^b^	*m/z* ^c^	
**1**	C14-SRR-NH_2_	1 2	627.47 314.24	627.64 314.50	16.55
**2**	C14-RSR-NH_2_	1 2	627.47 314.24	627.68 314.57	15.96
**3**	C14-RRS-NH_2_	1 2	627.47 314.24	627.64 314.55	15.91
**4**	C16-SRR-NH_2_	1 2	655.50 328.25	655.68 328.50	20.55
**5**	C16-RSR-NH_2_	1 2	655.50 328.25	655.71 328.65	19.93
**6**	C16-RRS-NH_2_	1 2	655.50 328.25	655.7 328.59	19.89
**7**	C18-SRR-NH_2_	1 2	683.52 342.27	683.73 342.66	24.78
**8**	C18-RSR-NH_2_	1 2	683.52 342.27	683.75 342.61	24.13
**9**	C18-RRS-NH_2_	1 2	683.52 342.27	683.72 342.6	24.04
**10**	C14-S(*GlcNAc*)RR-NH_2_	1 2	830.55 415.78	830.72 416.17	14.47
**11**	C14-RS(*GlcNAc*)R-NH_2_	1 2	830.55 415.78	830.79 416.22	14.82
**12**	C14-RRS(*GlcNAc*)-NH_2_	1 2	830.55 415.78	830.80 416.25	15.01
**13**	C16-S(*GlcNAc*)RR-NH2	1 2	858.58 429.79	858.80 430.06	18.26
**14**	C16-RS(*GlcNAc*)R-NH_2_	1 2	858.58 429.79	858.78 430.28	18.63
**15**	C16-RRS(*GlcNAc*)-NH_2_	1 2	858.58 429.79	858.82 430.34	18.80
**16**	C18-S(*GlcNAc*)RR-NH_2_	1 2	886.61 443.80	886.73 443.23	22.12
**17**	C18-RS(*GlcNAc*)R-NH_2_	1 2	886.61 443.80	886.81 444.27	22.50
**18**	C18-RRS(*GlcNAc*)-NH_2_	1 2	886.61 443.80	886.81 444.09	22.67

^a^—ion charge; ^b^—calculated mass to charge ratio; ^c^—measured mass to charge ratio.

**Table 2:** Antimicrobial activity (MIC [µg/mL]) of lipopeptides **1**–**18** against planktonic cells.

Peptide	Fatty Acid	*E.* *faecium* ATCC 700221	*S.* *aureus* ATCC 33591	*K.* *pneumoniae* ATCC 700603	*A.* *baumannii* ATCC BAA 1605	*P.* *aeruginosa* ATCC 9027	*K.* *aerogenes* ATCC 13048	*C.* *glabrata* ATCC 15126
Unmodified								
**1**	C14	16 *	16 *	128 *	32 *	32 *	128 *	32
**2**		8	8	512	64	64	512	32
**3**		8	16	512	64	64	512	32
**4**	C16	8	8	512	32	16	16	4
**5**		8	8	64	32	64	32	8
**6**		8	8	32	32	16	64	8
**7**	C18	8	16	16	32	64	16	4
**8**		8	16	32	64	64	32	4
**9**		8	8	16	32	64	32	4
Glycosylated								
**10**	C14	32	64	>512	128	256	>512	256
**11**		32	64	>512	64	128	>512	128
**12**		16	32	>512	64	256	>512	128
**13**	C16	16	16	**256**	32	32	512	32
**14**		16	16	128	32	32	64	16
**15**		8	16	256	32	64	256	16
**16**	C18	16	16	64	**16**	**32**	32	4
**17**		8	16	**16**	**16**	**32**	32	4
**18**		8	16	32	32	**32**	32	4

Bolded values indicate enhanced activity for gUSCLs in comparison to USCLs. * The analyses performed by our group in the previous study [22].

**Table 3:** Antibiofilm activity (MBEC [µg/mL]) of lipopeptides of **7**–**9** and **16**–**18**.

Peptide	Acid	*S.* *aureus*	*K.* *pneumoniae*	*A.* *baumannii*	*P.* *aeruginosa*	*C.* *glabrata*
Unmodified						
**7**	C18	512	512	512	512	128
**8**		256	512	512	>512	128
**9**		256	512	512	>512	128
Glycosylated						
**16**	C18	256	256	512	512	64
**17**		128	256	512	>512	64
**18**		128	128	256	512	128

**Table 4:** Hemolytic activity (HC_50_ [µg/mL]) and selectivity indexes of **1**–**18**.

Peptide	Acid	HC_50_	Selectivity Index (SI)						
			*E.* *faecium*	*S.* *aureus*	*K.* *pneumoniae*	*A.* *baumannii*	*P.* *aeruginosa*	*K.* *aerogenes*	*C.* *glabrata*
Unmodified									
**1**	C14	41.54 ± 1.29 *	2.60	2.60	0.32	1.30	1.30	0.32	1.30
**2**		186.64 ± 2.14	**23.33**	**23.33**	0.36	2.92	2.92	0.36	5.83
**3**		227.01 ± 5.69	**28.38**	**14.19**	0.44	3.55	3.55	0.44	7.09
**4**	C16	75.46 ± 0.73	9.43	9.43	0.15	2.36	4.72	4.72	**18.87**
**5**		82.21 ± 2.07	**10.28**	**10.28**	1.28	2.57	1.28	2.57	**10.28**
**6**		75.95 ± 1.57	9.49	9.49	2.37	2.37	4.75	1.19	9.49
**7**	C18	82.31 ± 1.36	**10.29**	5.14	5.14	2.57	1.29	5.14	**20.58**
**8**		335.12 ± 73.39	**41.89**	**20.95**	**10.47**	5.24	5.24	**10.47**	**83.78**
**9**		94.31 ± 2.64	**11.79**	**11.79**	5.89	2.95	1.47	2.95	**23.58**
Glycosylated									
**10**	C14	>512	**>16.00**	**>8.00**	-	4.00	>2.00	-	>2.00
**11**		364.17 ± 105.10	**11.38**	5.69	-	5.69	2.85	-	2.85
**12**		345.72 ± 51.94	**21.61**	**10.80**	-	5.40	1.35	-	2.70
**13**	C16	95.39 ± 2.40	5.96	5.96	0.37	2.98	2.98	0.19	2.98
**14**		73.34 ± 1.63	4.58	4.58	0.57	2.29	2.29	1.15	4.58
**15**		111.45 ± 11.65	**13.93**	6.97	0.44	3.48	1.74	0.44	6.97
**16**	C18	77.45 ± 1.13	4.84	4.84	1.21	4.84	2.42	2.42	**19.36**
**17**		45.25 ± 0.59	5.66	2.83	2.83	2.83	1.41	1.41	**11.31**
**18**		48.04 ± 2.45	6.01	3.00	1.50	1.50	1.50	1.50	**12.01**

Bold indicate SI equal or higher than 10. * Analyses were reported by our group in the previous study [22].

**Table 5:** Cytotoxicity (IC_50_ [µg/mL]) and selectivity indexes of **1**–**18**.

Peptide	Acid	IC_50_	Selectivity Index (SI)						
			*E.* *faecium*	*S.* *aureus*	*K.* *pneumoniae*	*A.* *baumannii*	*P.* *aeruginosa*	*K.* *aerogenes*	*C.* *glabrata*
Unmodified									
**1**	C14	1.41 ± 0.29	0.09	0.09	0.01	0.04	0.04	0.01	0.04
**2**		14.98 ± 2.05	**1.87**	**1.87**	0.03	0.23	0.23	0.03	0.47
**3**		14.27 ± 1.96	**1.78**	0.89	0.03	0.22	0.22	0.03	0.45
**4**	C16	4.02 ± 1.17	0.50	0.50	0.01	0.13	0.25	0.25	**1.01**
**5**		5.72± 2.27	0.72	0.72	0.09	0.18	0.09	0.18	0.72
**6**		7.84 ± 1.44	0.98	0.98	0.25	0.25	0.49	0.12	0.98
**7**	C18	8.28 ± 1.18	**1.04**	0.52	0.52	0.26	0.13	0.52	**2.07**
**8**		7.97 ± 1.51	1.00	0.50	0.25	0.12	0.12	0.25	**1.99**
**9**		10.68 ± 1.61	**1.34**	**1.34**	0.67	0.33	0.17	0.33	**2.67**
Glycosylated									
**10**	C14	32.12 ± 6.26	1.00	0.50	-	0.25	0.13	-	0.13
**11**		25.16 ± 3.47	0.79	0.39	-	0.39	0.20	-	0.20
**12**		20.46 ± 3.05	**1.28**	0.64	-	0.32	0.08	-	0.16
**13**	C16	10.61 ± 2.04	0.66	0.66	0.04	0.33	0.33	0.02	0.33
**14**		14.08 ± 3.48	0.88	0.88	0.11	0.44	0.44	0.22	0.88
**15**		5.16 ± 1.32	0.65	0.32	0.02	0.16	0.08	0.02	0.32
**16**	C18	10.12 ± 0.66	0.63	0.63	0.16	0.63	0.32	0.32	**2.53**
**17**		9.45 ± 0.89	**1.18**	0.59	0.59	0.59	0.30	0.30	**2.36**
**18**		13.85 ± 1.71	**1.73**	0.87	0.43	0.43	0.43	0.43	**3.46**

Bold indicate SI above 1.

## Data Availability

Not applicable.

## Supplementary Materials

The following supporting information can be downloaded at: https://www.mdpi.com/article/10.3390/biom13010172/s1, Figure S1: Log_2_MIC vs. adjusted retention time for *E. fecium*.; Figure S2: Log_2_MIC vs. adjusted retention time for *S. aureus*.; Figure S3: Log_2_MIC vs. adjusted retention time for *K. pneumoniae.*; Figure S4: Log_2_MIC vs. adjusted retention time for *A. baumannii.*; Figure S5: Log_2_MIC vs. adjusted retention time for *P. aeruginosa*.; Figure S6: Log_2_MIC vs. adjusted retention time for *K. aerogenes*.; Figure S7: Log_2_MIC vs. adjusted retention time for *C. glabrata*.; Figure S8: Log_2_SI (HC_50_) vs. adjusted retention time for *E. fecium*.; Figure S9: Log_2_SI (HC_50_) vs. adjusted retention time for *S. aureus*.; Figure S10: Log_2_SI (HC_50_) vs. adjusted retention time for *K. pneumoniae.*; Figure S11: Log_2_SI (HC_50_) vs. adjusted retention time for *A. baumannii*.; Figure S12: Log_2_SI (HC_50_) vs. adjusted retention time for *P. aeruginosa.;* Figure S13: Log_2_SI (HC_50_) vs. adjusted retention time for *K. aerogenes.;* Figure S14: Log_2_SI (HC_50_) vs. adjusted retention time for *C. glabrata.;* Figure S15: Log_2_SI (IC_50_) vs. adjusted retention time for *E. fecium.;* Figure S16: Log_2_SI (IC_50_) vs. adjusted retention time for *S. aureus.;* Figure S17: Log_2_SI (IC_50_) vs. adjusted retention time for *K. pneumoniae.;* Figure S18: Log_2_SI (IC_50_) vs. adjusted retention time for *A. baumannii.;* Figure S19: Log_2_SI (IC_50_) vs. adjusted retention time for *P. aeruginosa.;* Figure S20: Log_2_SI (iC_50_) vs. adjusted retention time for *K. aerogenes.;* Figure S21: Log_2_SI (IC_50_) vs. adjusted retention time for *C. glabrata.*
