# Supplementary material for: Glycosylated Lipopeptides—Synthesis and Evaluation of Antimicrobial Activity and Cytotoxicity
Source: Biomolecules. 2023 Jan 13;13(1):172. doi: 10.3390/biom13010172 (PMC9855884; doi:10.3390/biom13010172)
Supplement: Supplementary file 1 [file biomolecules-13-00172-s001.zip › biomolecules-2131987-supplementary.pdf]

Supplementary materials

Glycosylated lipopeptides – synthesis and evaluation of anti-microbial activity and cytotoxicity

Log_2MIC *vs* adjusted retention time

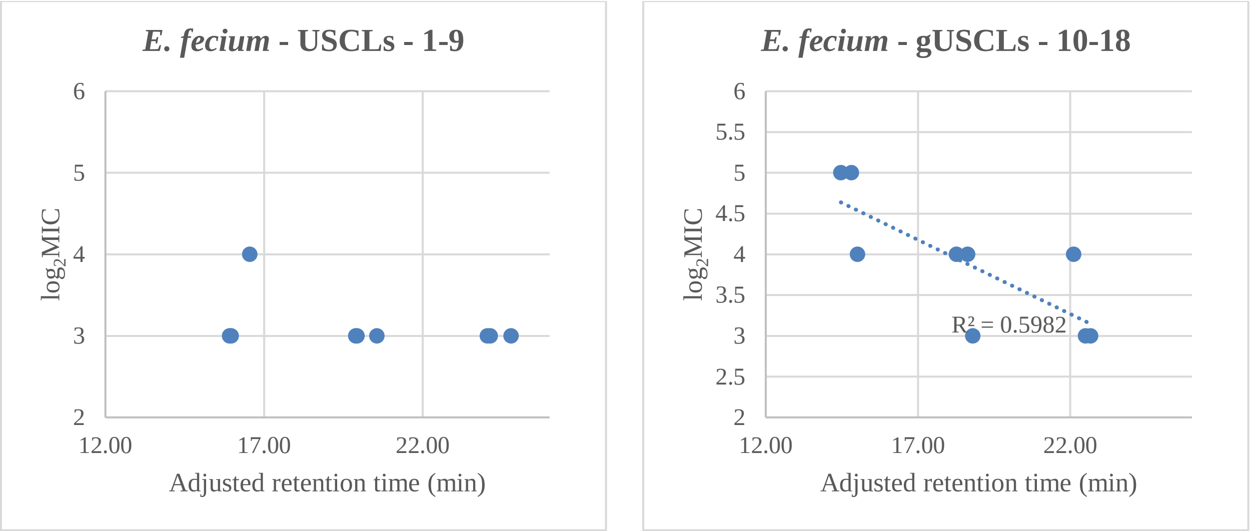

Figure S1. Log_2MIC *vs* adjusted retention time for *E. fecium.*

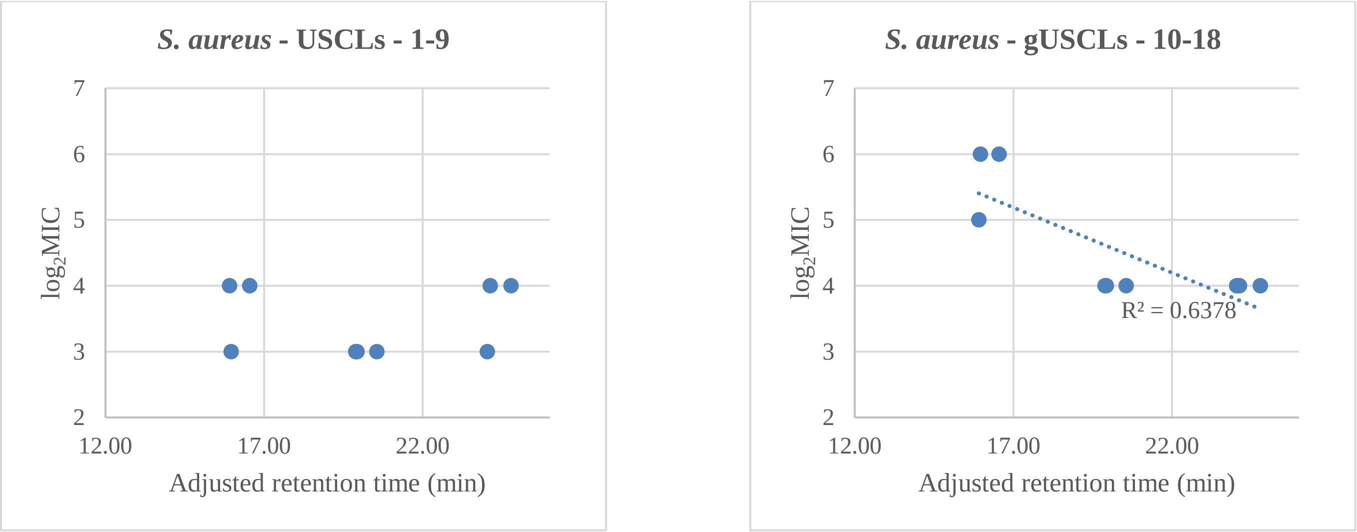

Figure S2. Log_2MIC *vs* adjusted retention time for *S. aureus.*

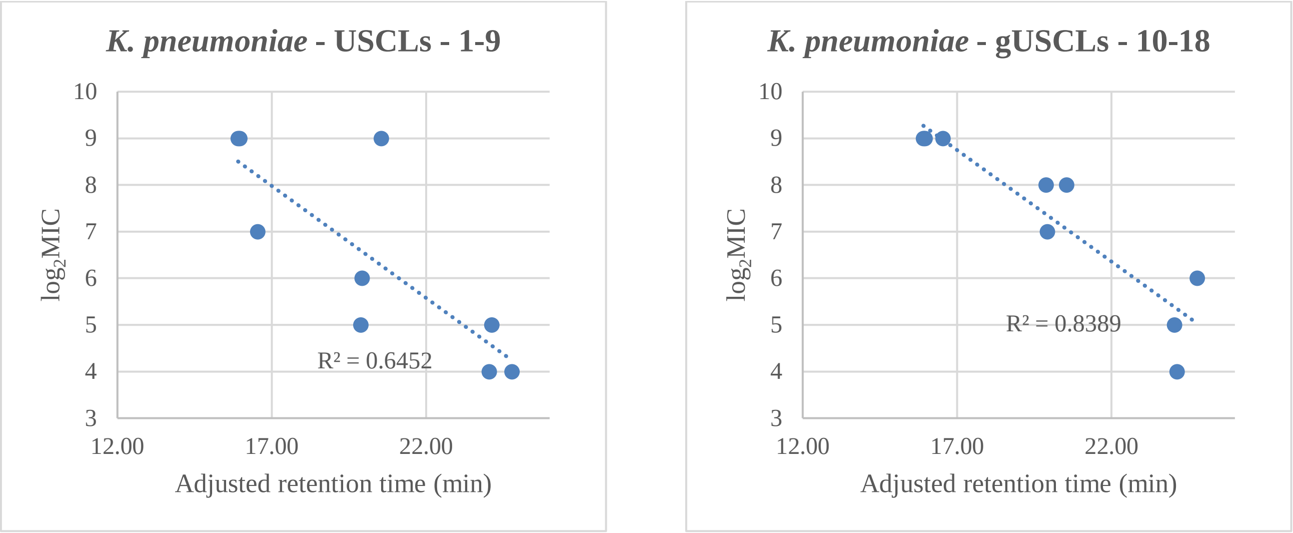

Figure S3. Log2MIC *vs* adjusted retention time for *K. pneumoniae.*

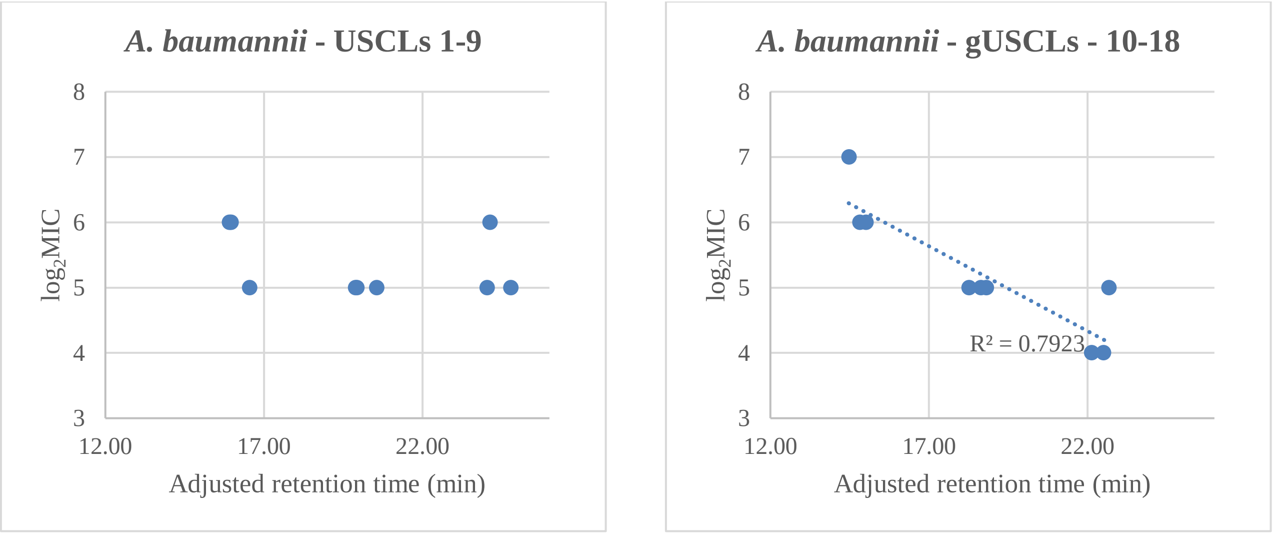

Figure S4. Log2MIC *vs* adjusted retention time for *A. baumannii.*

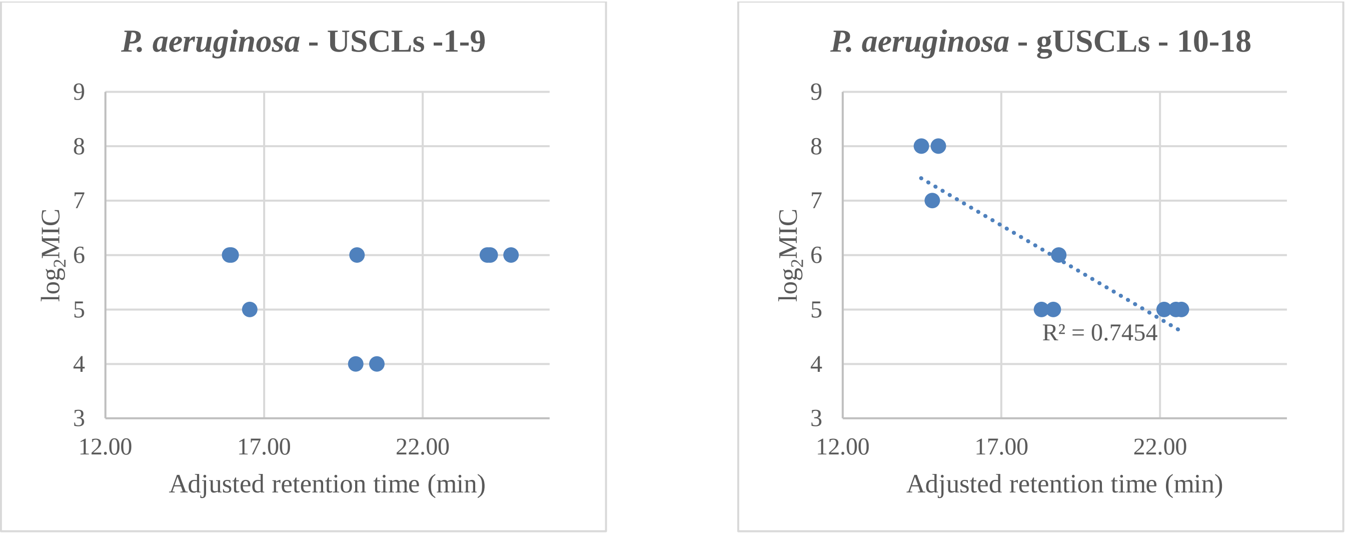

Figure S5. Log2MIC *vs* adjusted retention time for *P. aeruginosa.*

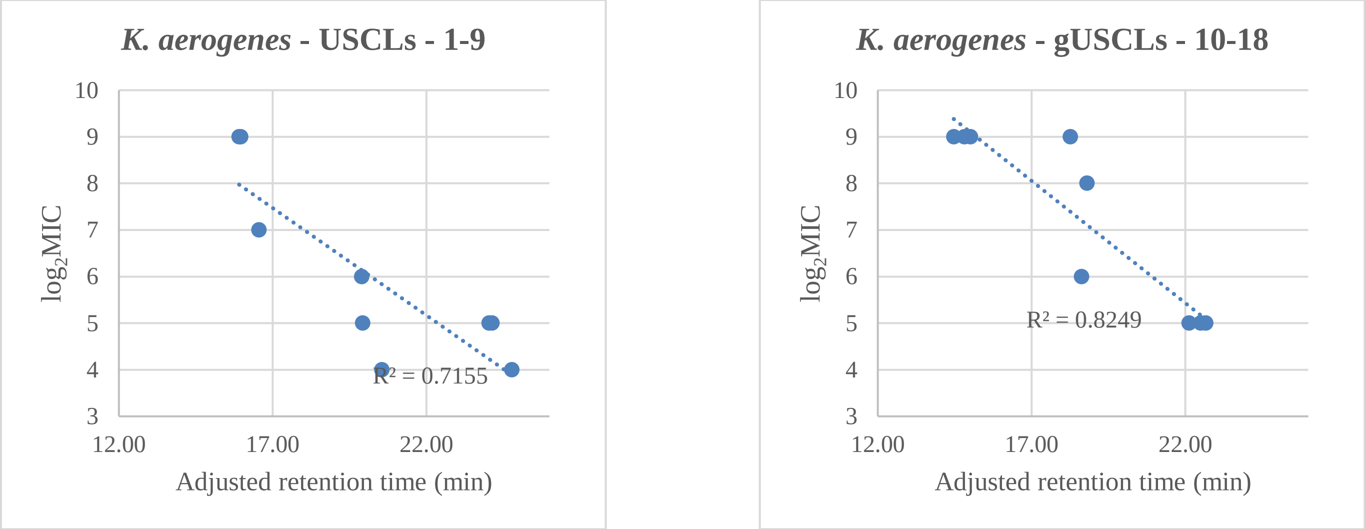

Figure S6. Log2MIC *vs* adjusted retention time for *K. aerogenes.*

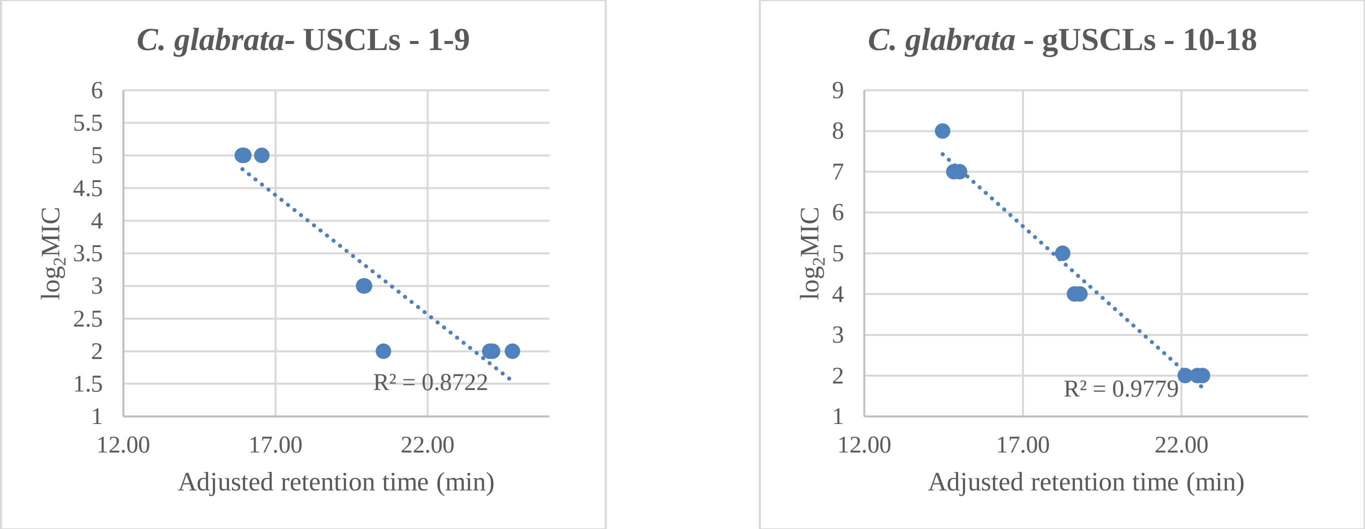

Figure S7. Log2MIC *vs* adjusted retention time for *C. glabrata.*

Log$_2$SI (HC$_{50}$) *vs* adjusted retention time

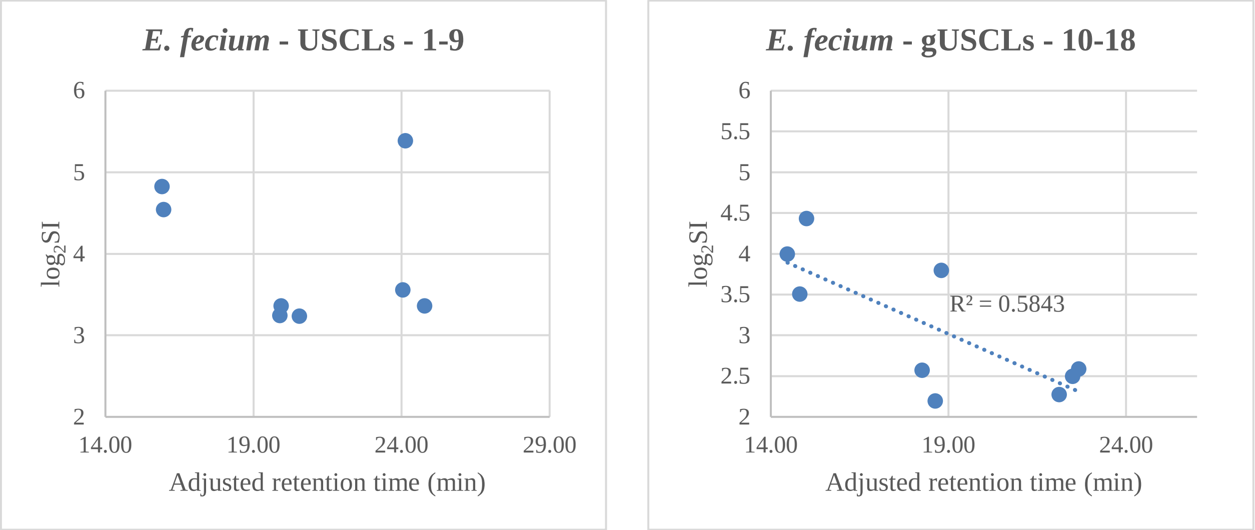

Figure S8. Log$_2$SI (HC$_{50}$) *vs* adjusted retention time for *E. fecium.*

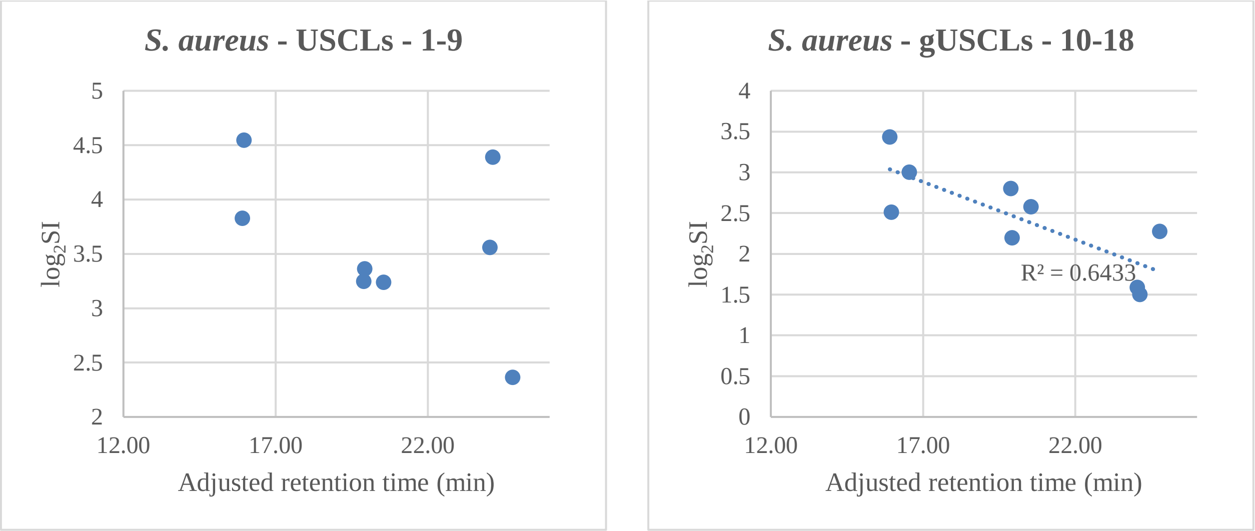

Figure S9. Log$_2$SI (HC$_{50}$) *vs* adjusted retention time for *S. aureus.*

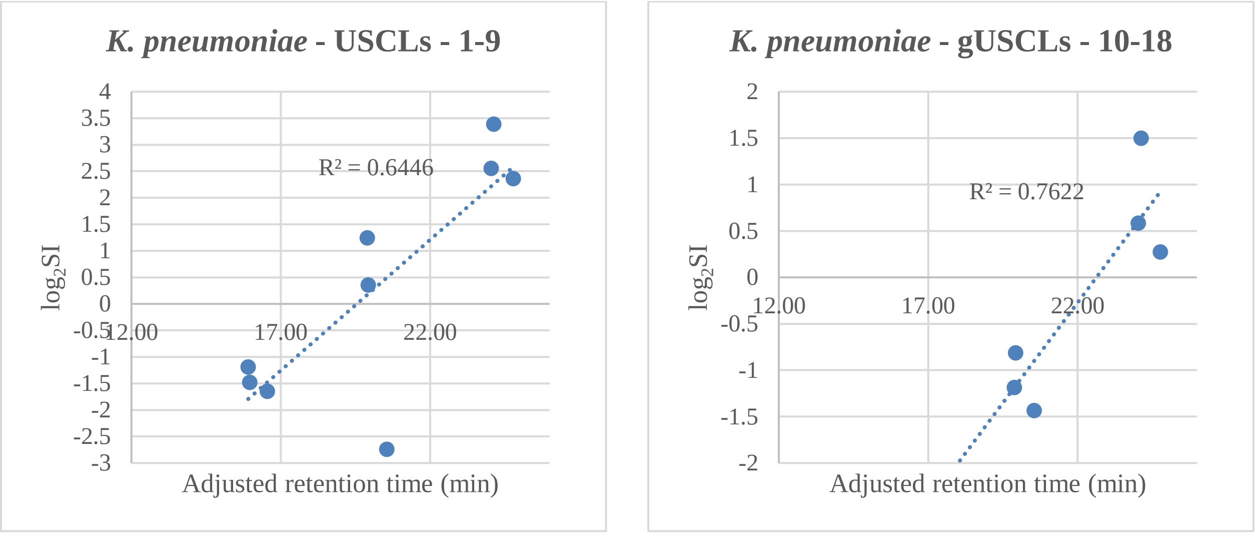

Figure S10. Log_2SI (HC_{50}) *vs* adjusted retention time for *K. pneumoniae.*

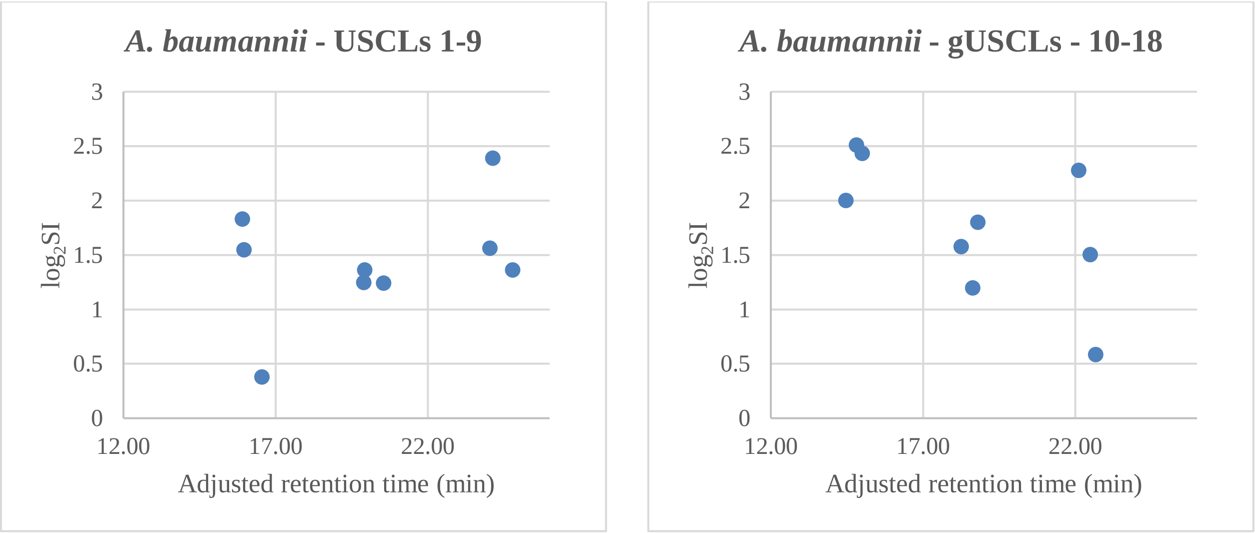

Figure S11. Log_2SI (HC_{50}) *vs* adjusted retention time for *A. baumannii.*

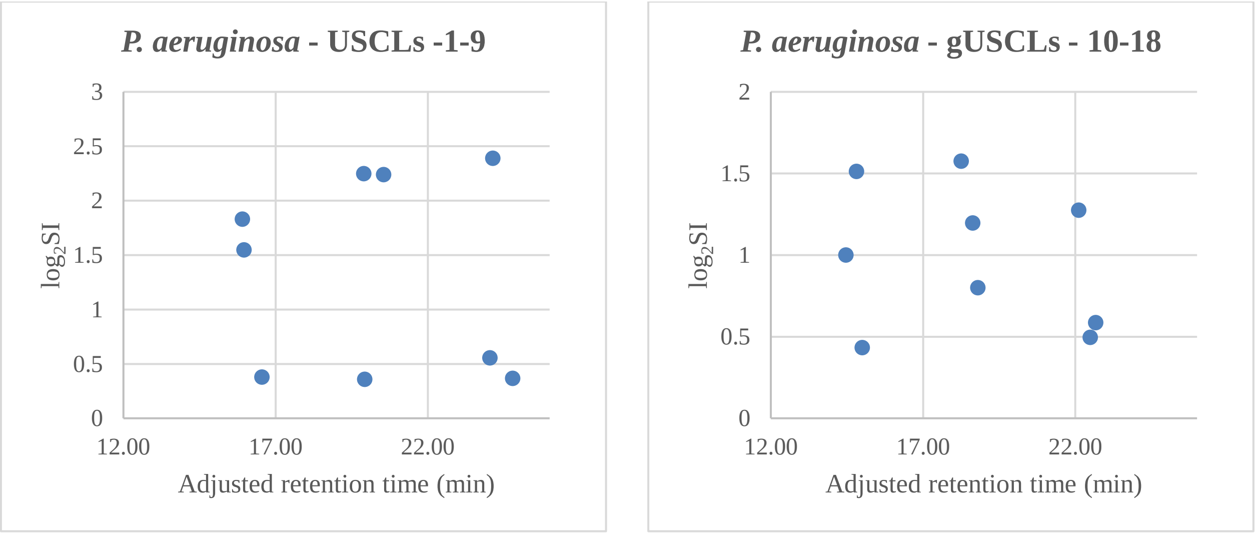

Figure S12. Log2SI (HC50) *vs* adjusted retention time for *P. aeruginosa*.

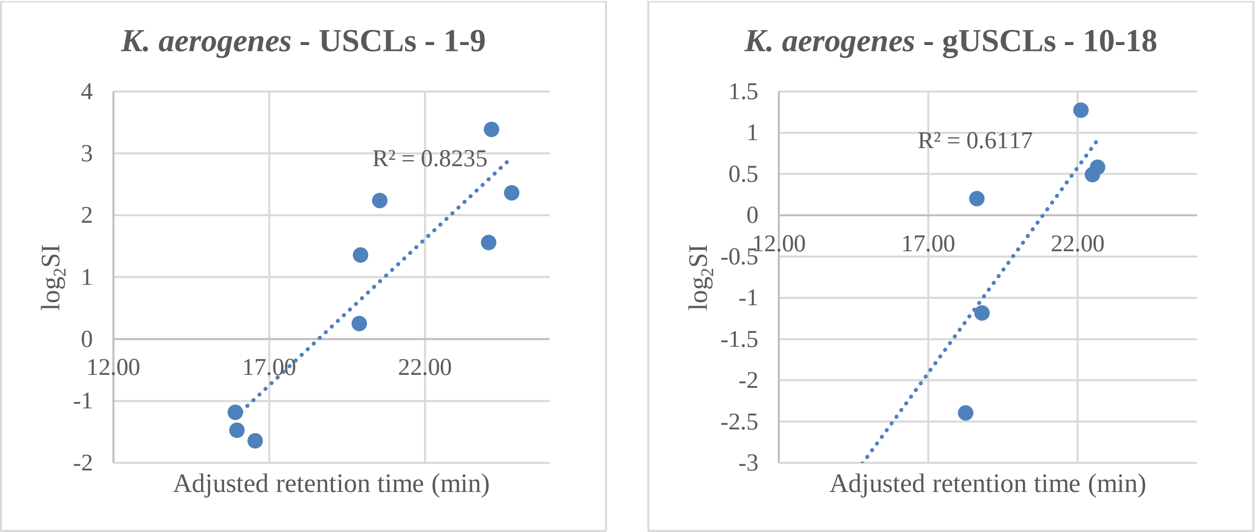

Figure S13. Log2SI (HC50) *vs* adjusted retention time for *K. aerogenes*.

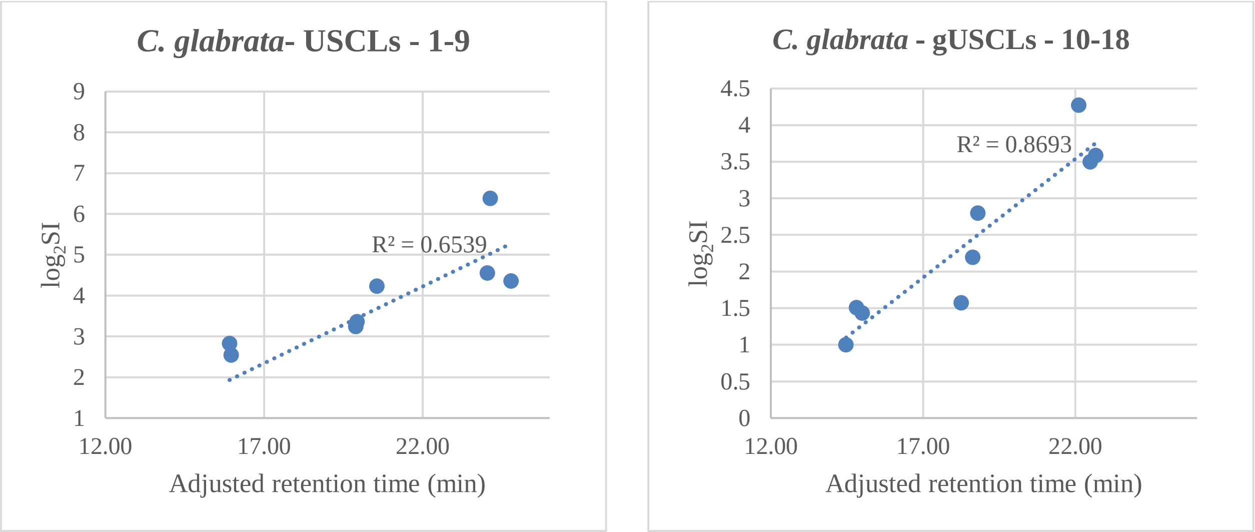

Figure S14. Log2SI (HC50) *vs* adjusted retention time for *C. glabrata*.

Log2SI (IC50) *vs* adjusted retention time

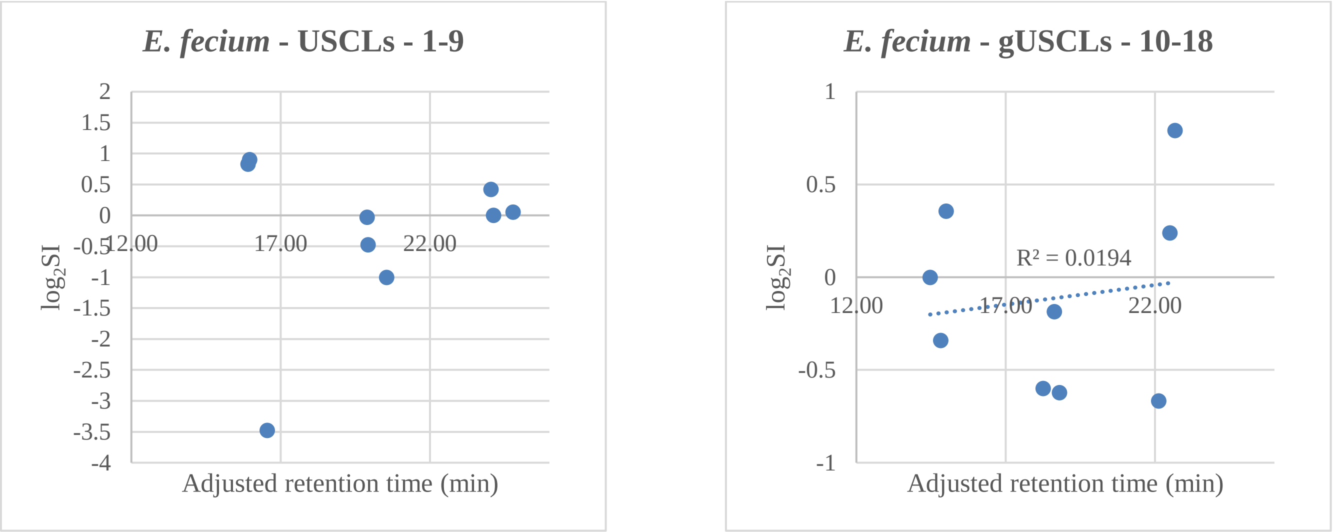

Figure S15. Log2SI (IC50) *vs* adjusted retention time for *E. fecium*.

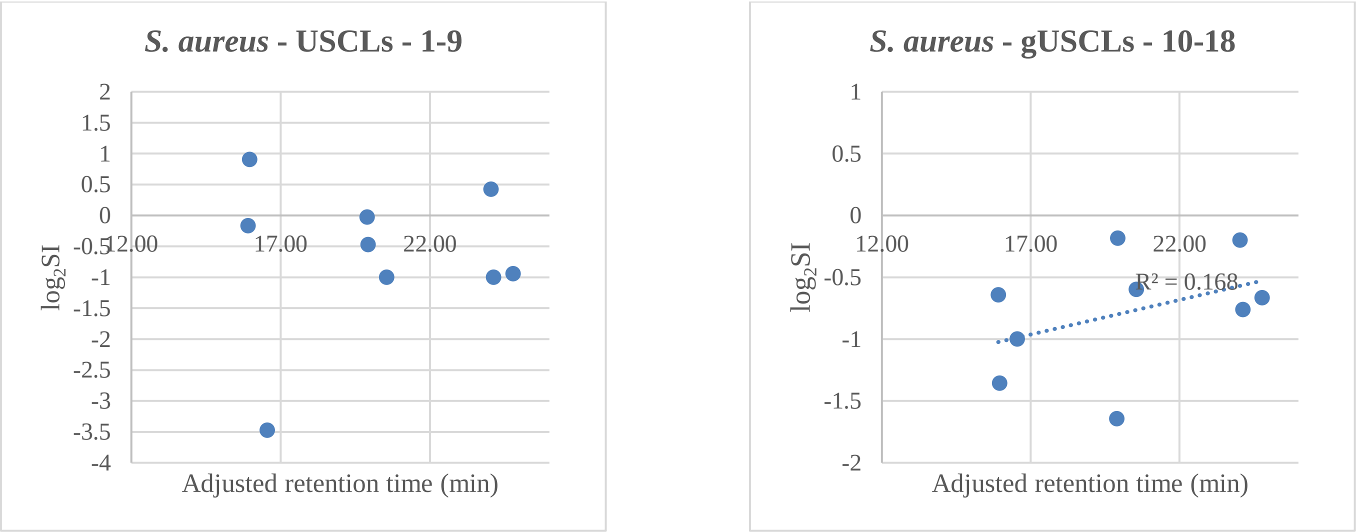

Figure S16. Log2SI (IC50) *vs* adjusted retention time for *S. aureus*.

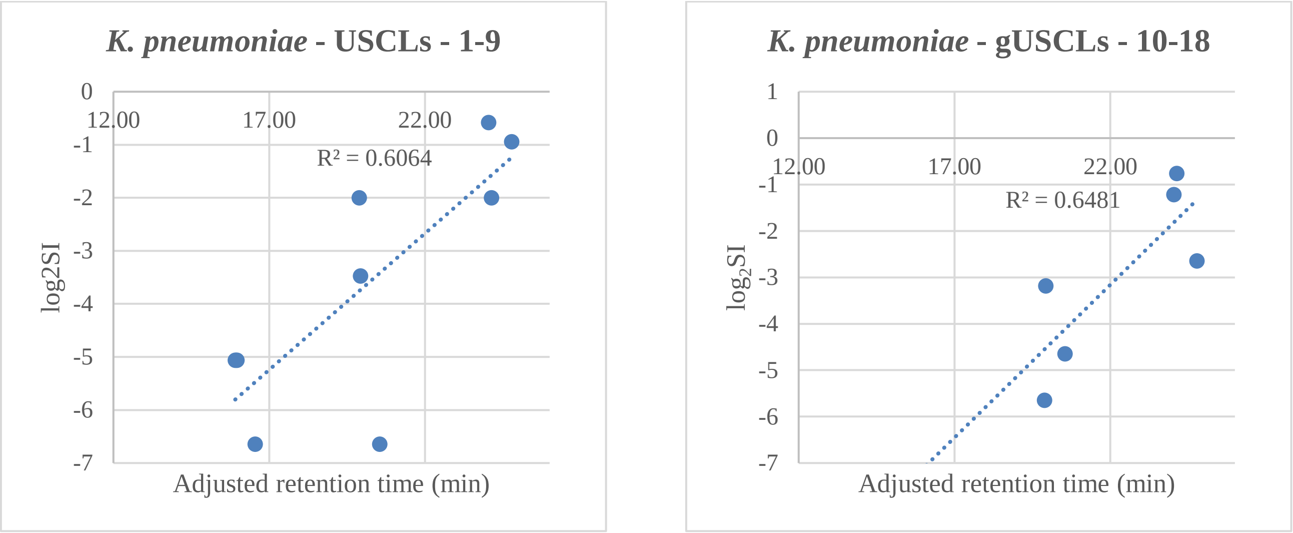

Figure S17. Log2SI (IC_{50}) *vs* adjusted retention time for *K. pneumoniae.*

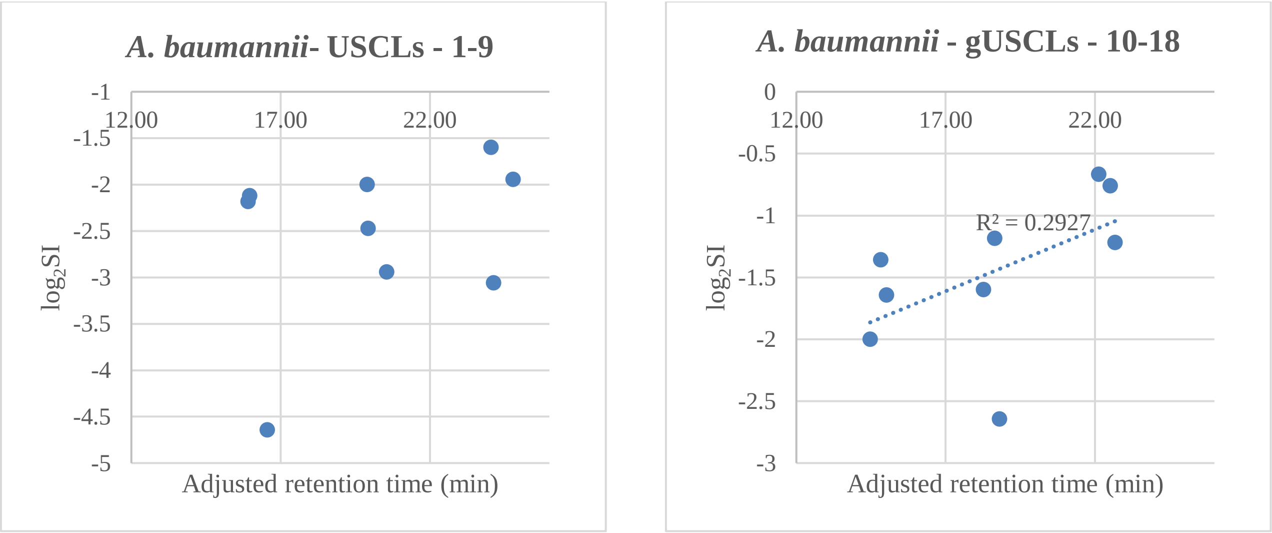

Figure S18. Log2SI (IC_{50}) *vs* adjusted retention time for *A. baumannii.*

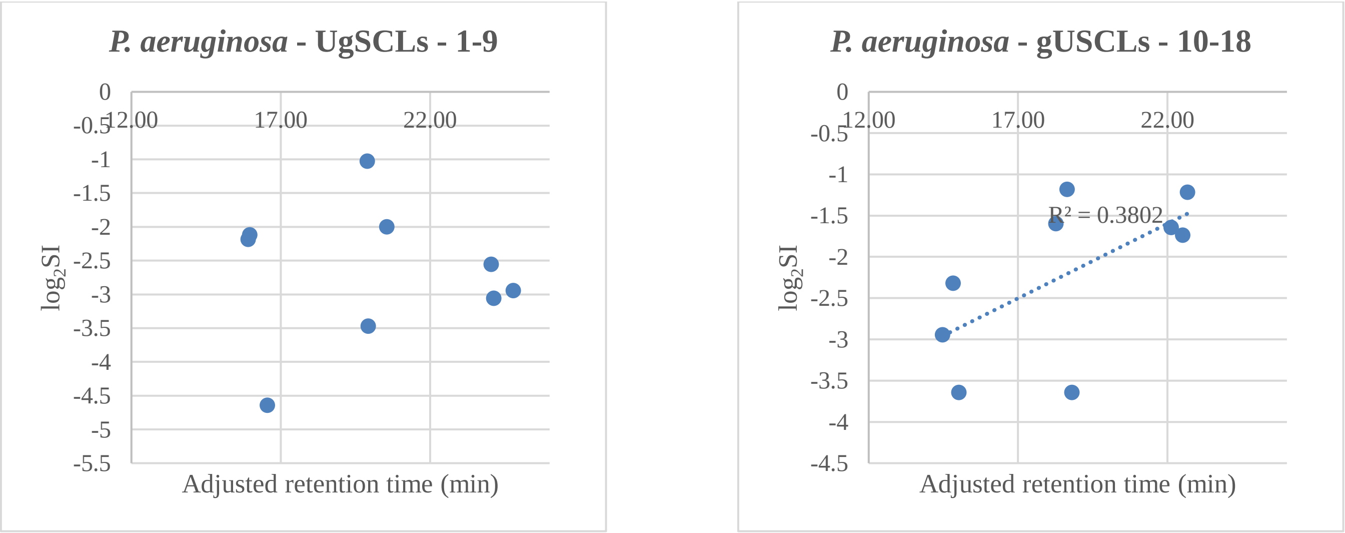

Figure S19. Log2SI (IC50) *vs* adjusted retention time for *P. aeruginosa.*

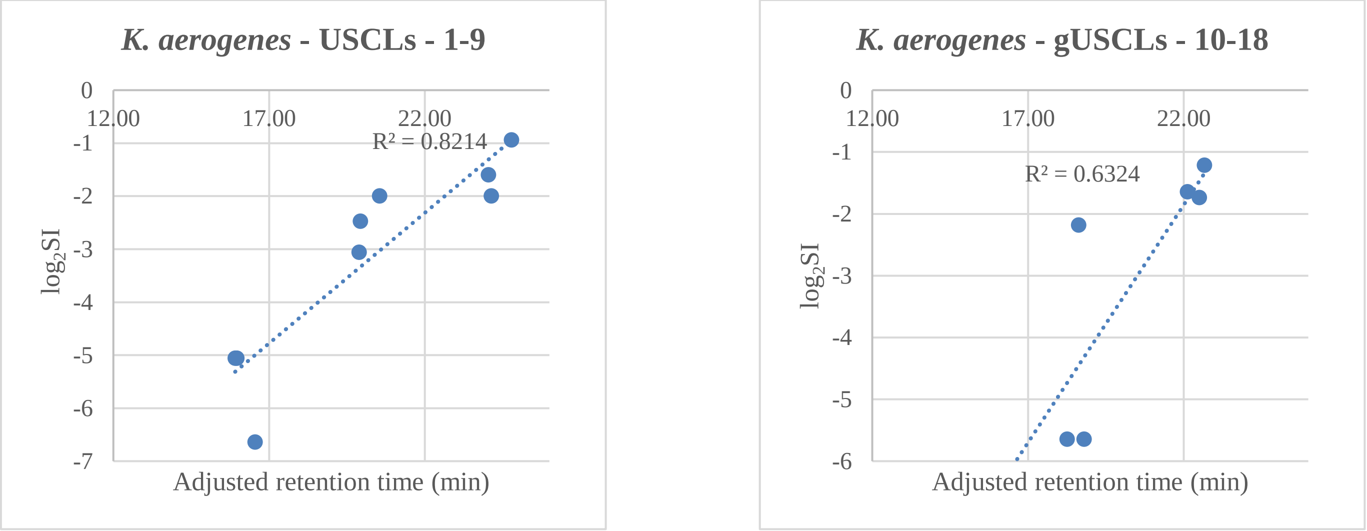

Figure S20. Log2SI (IC50) *vs* adjusted retention time for *K. aerogenes.*

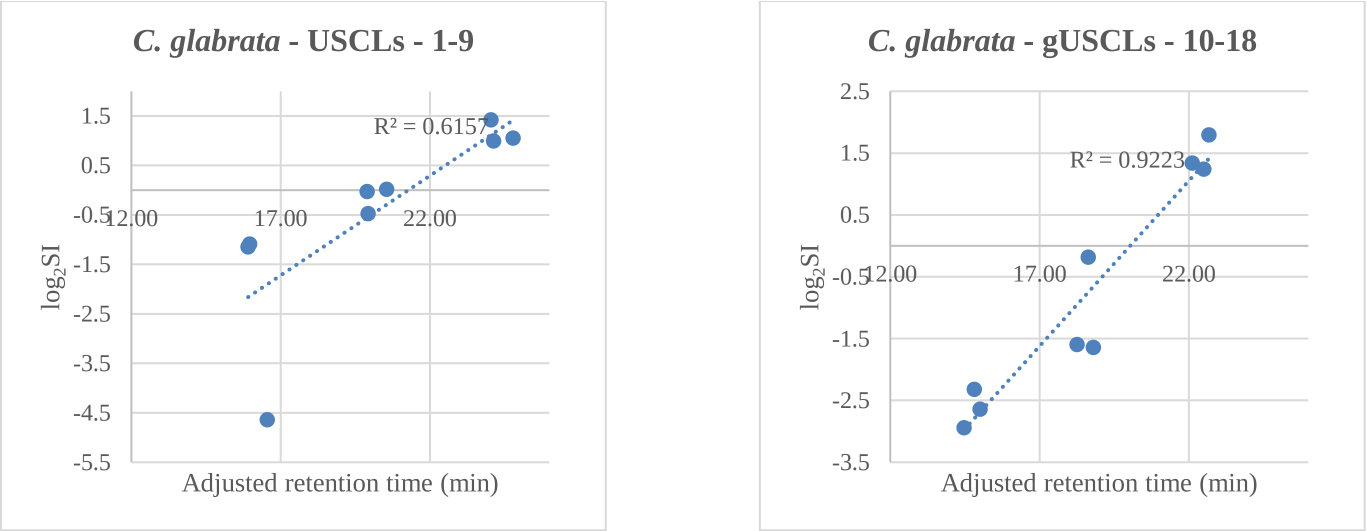

Figure S21. Log2SI (IC50) *vs* adjusted retention time for *C. glabrata.*
